# Supplementary material for: Stability and nuclear localization of yeast telomerase depend on protein components of RNase P/MRP
Source: Nat Commun. 2020 May 1;11:2173. doi: 10.1038/s41467-020-15875-9 (PMC7195438; doi:10.1038/s41467-020-15875-9)
Supplement: Supplementary file 1 — Supplementary Information [file 41467_2020_15875_MOESM1_ESM.pdf]

Stability and nuclear localization of yeast telomerase depend on protein components of RNase P/MRP

Garcia et al.

Supplementary information

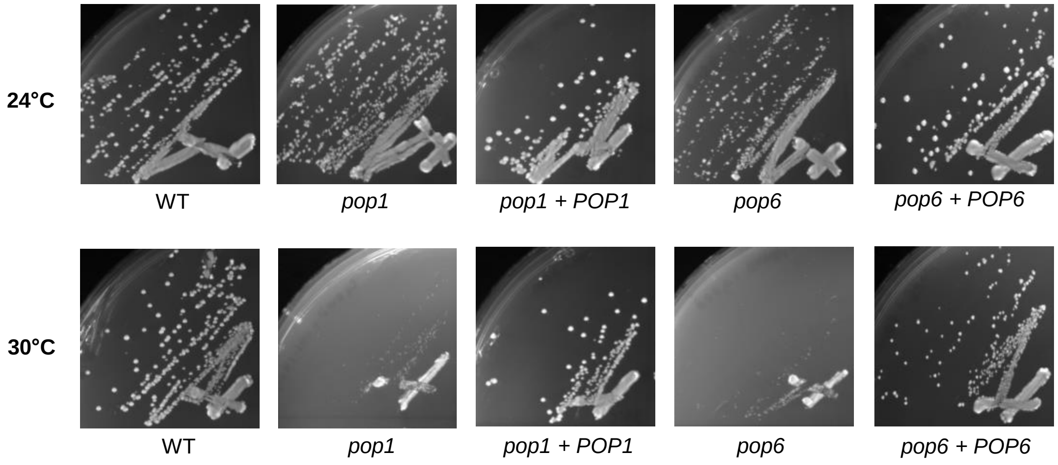

Supplementary Figure 1: Growth rates of *pop* cells are comparable to WT at 24°C.

A plasmid borne copy of *POP1* (or *POP6*) suppresses the growth defects of *pop1* (or *pop6*) cells. Cells were grown for ~50 generations at the indicated temperature on YC plates. Images were taken after 4 days at 24°C or 2 days at 30°C. For growth rates, see Fig. 2C, D.

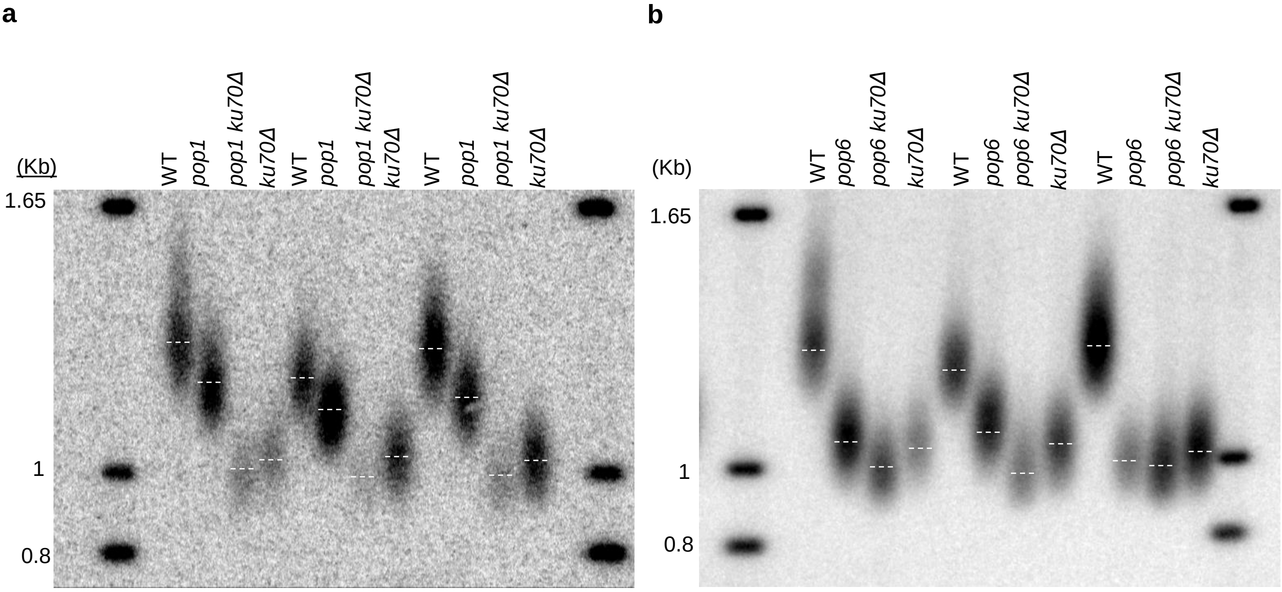

c

Average telomere length.

Strain	Average length (bp)	± bp	P value relative to *pop1/pop6*	P value relative to *ku70Δ*
**WT**	392	38		
pop1	285	25		
pop6	175	30		
ku70Δ	167	14		
pop1 ku70Δ	143	4	1.8 $\times 10^{-8}$	5.3 $\times 10^{-4}$
pop6 ku70Δ	142	2	1.0 $\times 10^{-3}$	7.4 $\times 10^{-4}$

Supplementary Figure 2: *ku70Δ* and *pop* mutations affect telomere length by different mechanisms.

Cells were grown at 30°C for ~75 generations prior to DNA extraction. Telomere lengths were determined as described for Fig. 3. **a)** WT, *pop1*, *ku70Δ* and *pop1 ku70Δ* and **b)** WT, *pop6*, *ku70Δ* and *pop6 ku70Δ*. Sizes of molecular weight standards are shown. White dotted lines mark the average telomere length in each sample**. c)** Quantitation of telomere lengths in at least three biological isolates for each strain was done as described for Fig 3. *P* values are indicated relative to *pop1*, *pop6* or *ku70Δ* cells and were calculated using unpaired two-tailed Student's *t*-test.

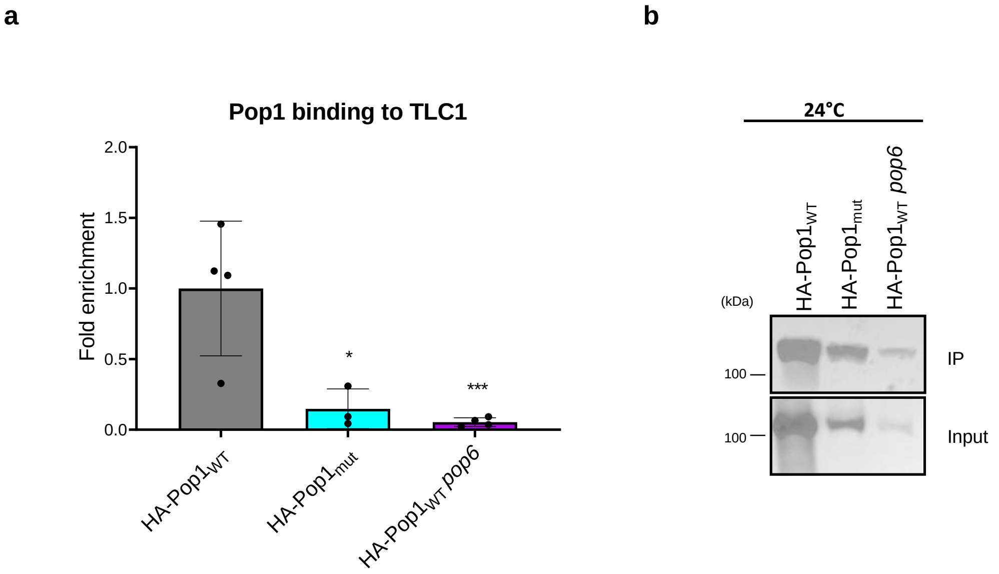

Supplementary Figure 3: Pop1 binding to TLC1 RNA is deficient in *pop1* and *pop6* cells at 24°C.

RNA immunoprecipitations were done for HA-Pop1$_{WT}$ (WT cells), HA-Pop1$_{mut}$ *(pop1* cells*)* and HA-Pop1$_{WT}$ (*pop6* cells) grown at 24°C for ~50 generations. **a)** Binding of HA-Pop1$_{WT}$ or HA-Pop1$_{mut}$ to TLC1 RNA was measured by RT-qPCR. RNA association was calculated by IP/INPUT of TLC1 RNA and normalized to IP/INPUT ACT1 mRNA. The TLC1/ACT1 average in WT was defined as one. Fold enrichment and significance in *pop1* and *pop6* cells is relative to WT. **b)** Representative western blots of HA-Pop1$_{WT}$, HA-Pop1$_{mut}$ and HA-Pop1$_{WT}$ *pop6* in IP and INPUT samples.

Error bars are one standard deviation from the average value of three or more independent experiments, P-values were calculated using unpaired two-tailed Student's *t*-test; * indicates $P \leq 0.05$, ** $P \leq 0.01$, *** $P \leq 0.001$, ****, $P \leq 0.0001$; NS, not significant, $P > 0.05$. Source data are provided as a Source Data file.

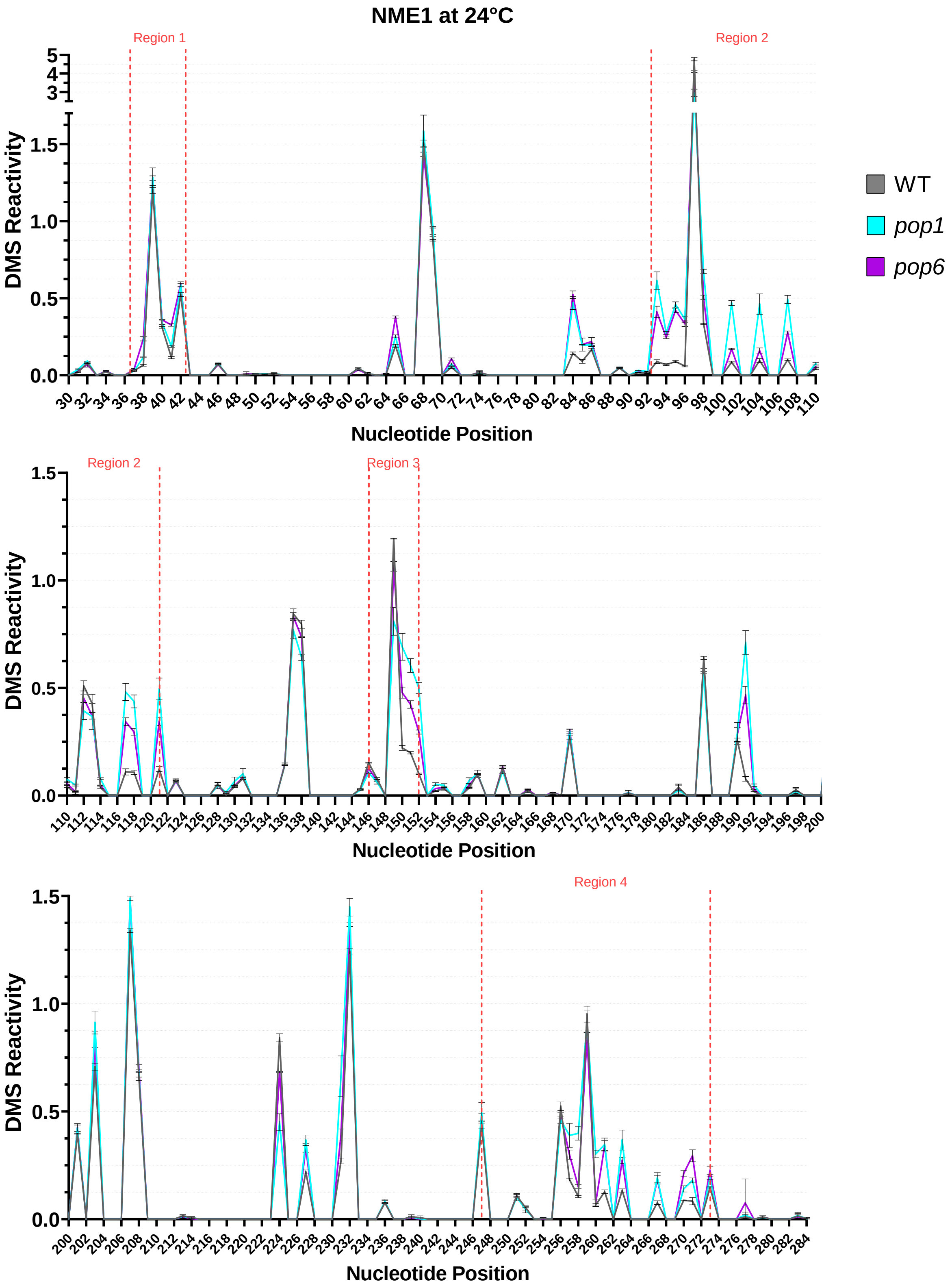

Supplementary Figure 4: The structure of NME1 RNA is affected in *pop* cells at 24°C

DMS-MaPseq differential reactivity analysis from strains grown at 24°C for ~50 generations. Normalized DMS reactivity profiles of all analyzed regions (nts 30-284) in NME1 RNA. DMS reactivities are shown for WT (gray), *pop1* (blue) and *pop6* (purple). DMS reactivities from three independent biological replicates were averaged and the standard deviations are shown. Regions marked by red dashes had statistically significant differential DMS reactivity between the WT and *pop* cells (shown in Fig. 8).

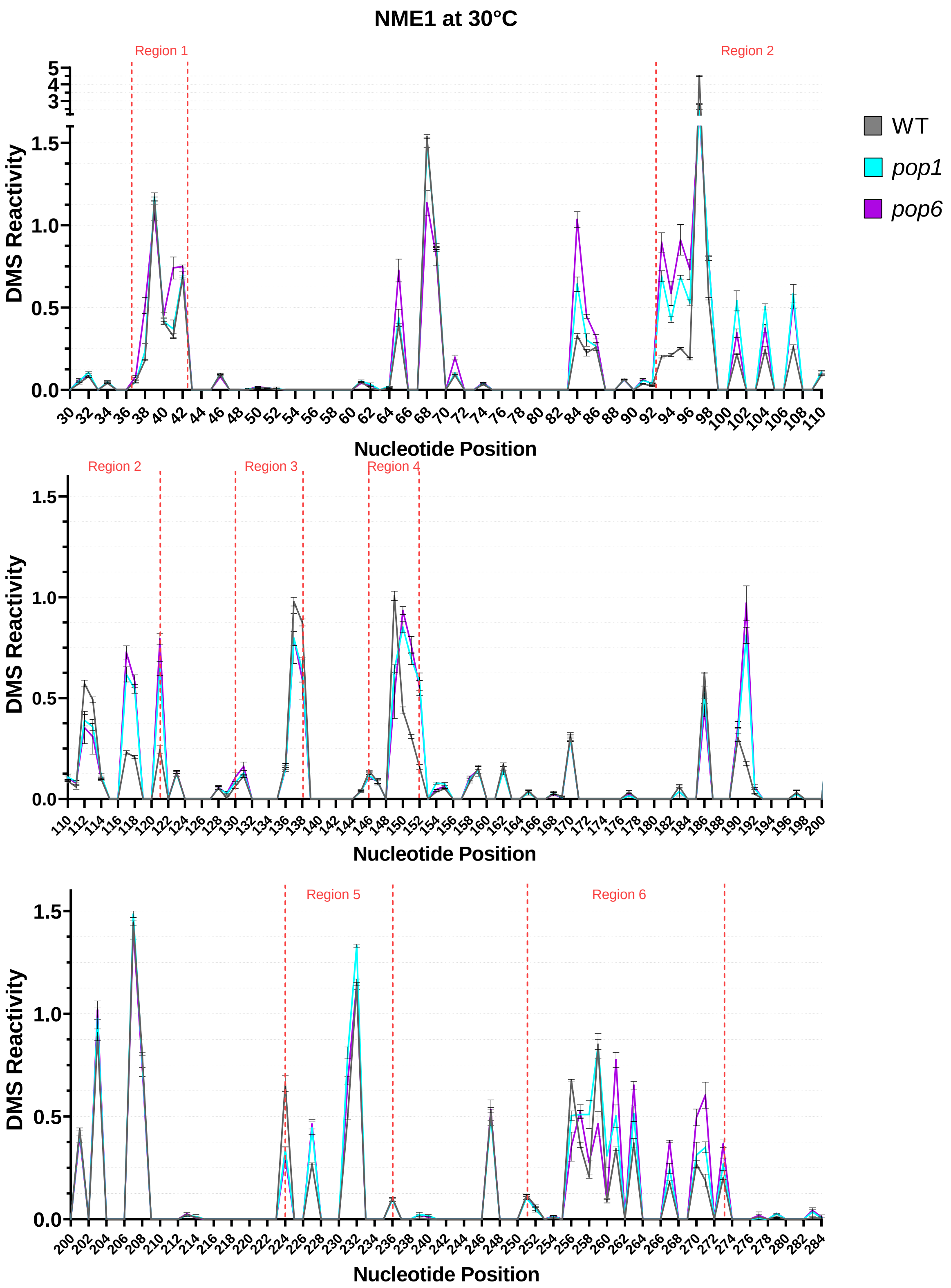

Supplementary Figure 5: The structure of NME1 RNA is globally affected in *pop* cells at 30°C.

Normalized DMS reactivity profiles of all the regions analyzed (nts 30-284) in NME1 RNA in cells grown at 30°C for ~50 generations. Red dashes indicate the regions with statistically significant differential DMS reactivity between WT and *pop* cells (shown in Supp. Fig 6).

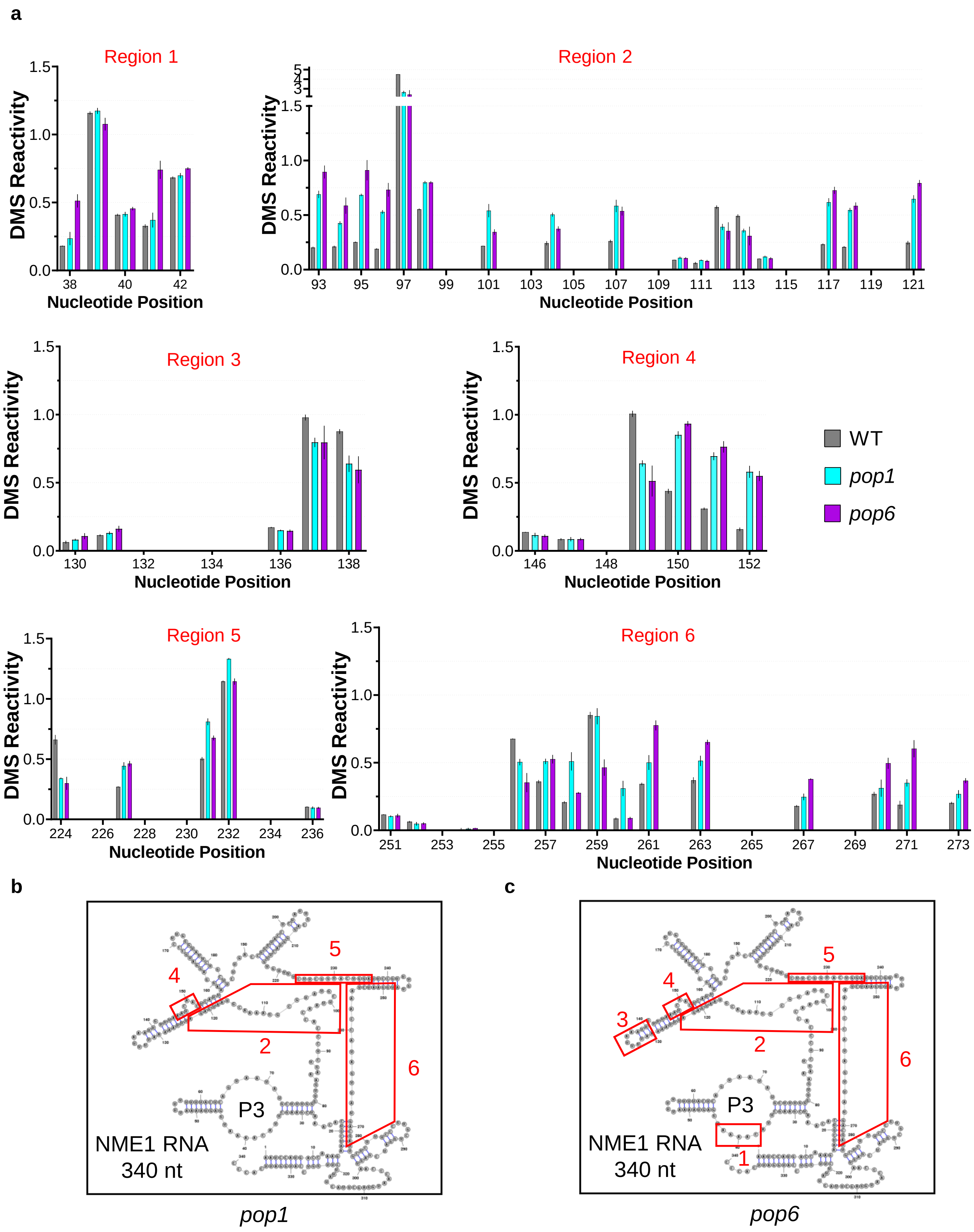

Supplementary Figure 6: Regions affected in the NME1 RNA of *pop* cells at 30°C

a) Bar graphs indicate the average normalized DMS reactivity in NME1 RNA of the 6 statistically significant regions shown in Supplementary Fig. 5. Error bars were calculated from the standard deviation of three biological replicates. Regions shown in A) are mapped in the NME1 RNA structure for **b)** *pop1* and **c)** *pop6* cells.

q values and Δ*d* values for each region in NME1 in cells grown at 30°C

		pop1	***pop6***
**Region 1**	q-value	NS	0.05
	Δ*d*	NS	0.12
**Region 2**	q-value	0.0002	0.0002
	Δ*d*	0.18	0.18
**Region 3**	q-value	NS	0.054
	Δ*d*	NS	0.06
**Region 4**	q-value	0.054	0.054
	Δ*d*	0.16	0.06
**Region 5**	q-value	0.054	0.098
	Δ*d*	0.11	0.09
**Region 6**	q-value	0.006	0.0015
	Δ*d*	0.08	0.13

Supplementary Figure 7: Changes in six regions of NME1 RNA of *pop* cells at 30°C are statistically significant.

Q-values and Δ*d* values of regions 2,4,5, and 6 in *pop1* and regions 1-6 in *pop6* compared to WT are shown. NME1 RNA regions are indicated in Supp. Fig 5 and Supp Fig. 6.

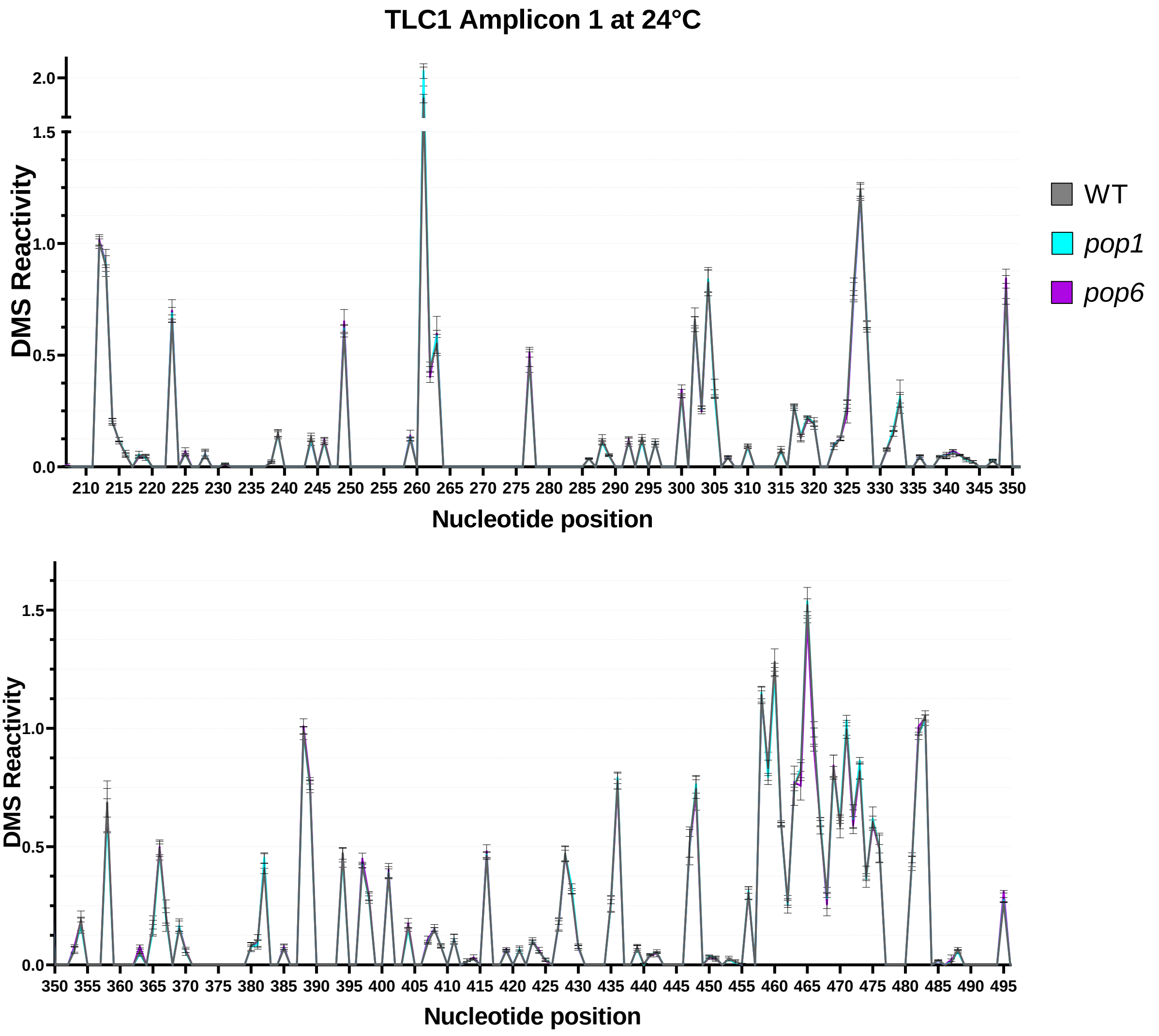

Supplementary Figure 8: DMS reactivity profiles in TLC1 amplicon 1 at 24°C

Normalized DMS reactivity profiles of the TLC1 amplicon 1 (nts 212-495) in cells grown at 24°C for ~50 generations. The WT (gray), *pop1* (blue) and *pop6* (purple) DMS reactivities are all shown in the line graph, but they are too similar to be differentiated. No region in amplicon one was significantly different in WT versus *pop1* or *pop6* cells.

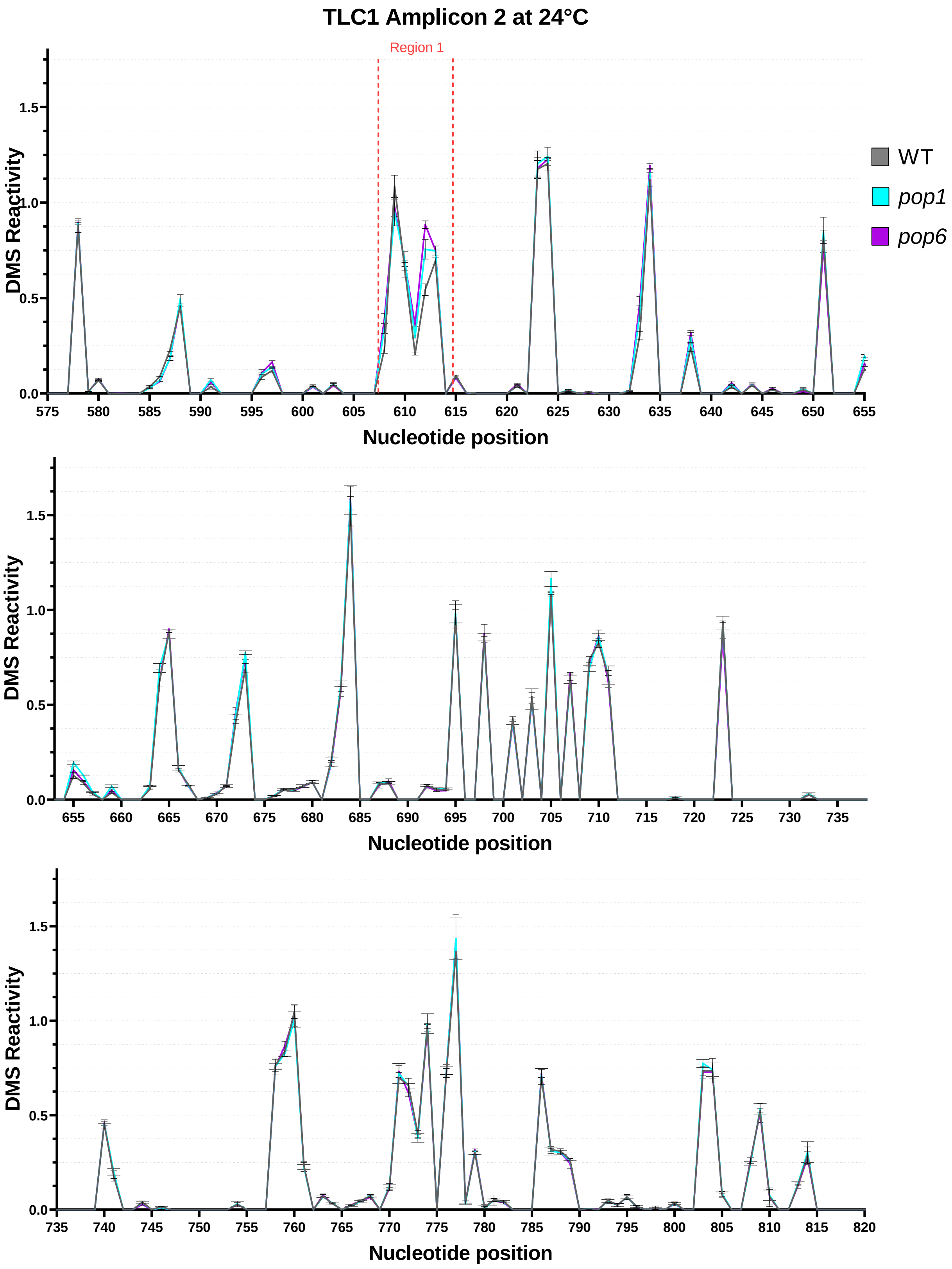

Supplementary Figure 9: DMS reactivity profiles in TLC1 amplicon 2 at 24°C

Normalized DMS reactivity profiles of the TLC1 amplicon 2 (nts 578-814) in cells grown at 24°C for ~50 generations. Only one region shown by red dashes had a statistically significant difference in reactivity in *pop* compared to WT cells. A higher resolution of the region is shown in Fig. 8D, E.

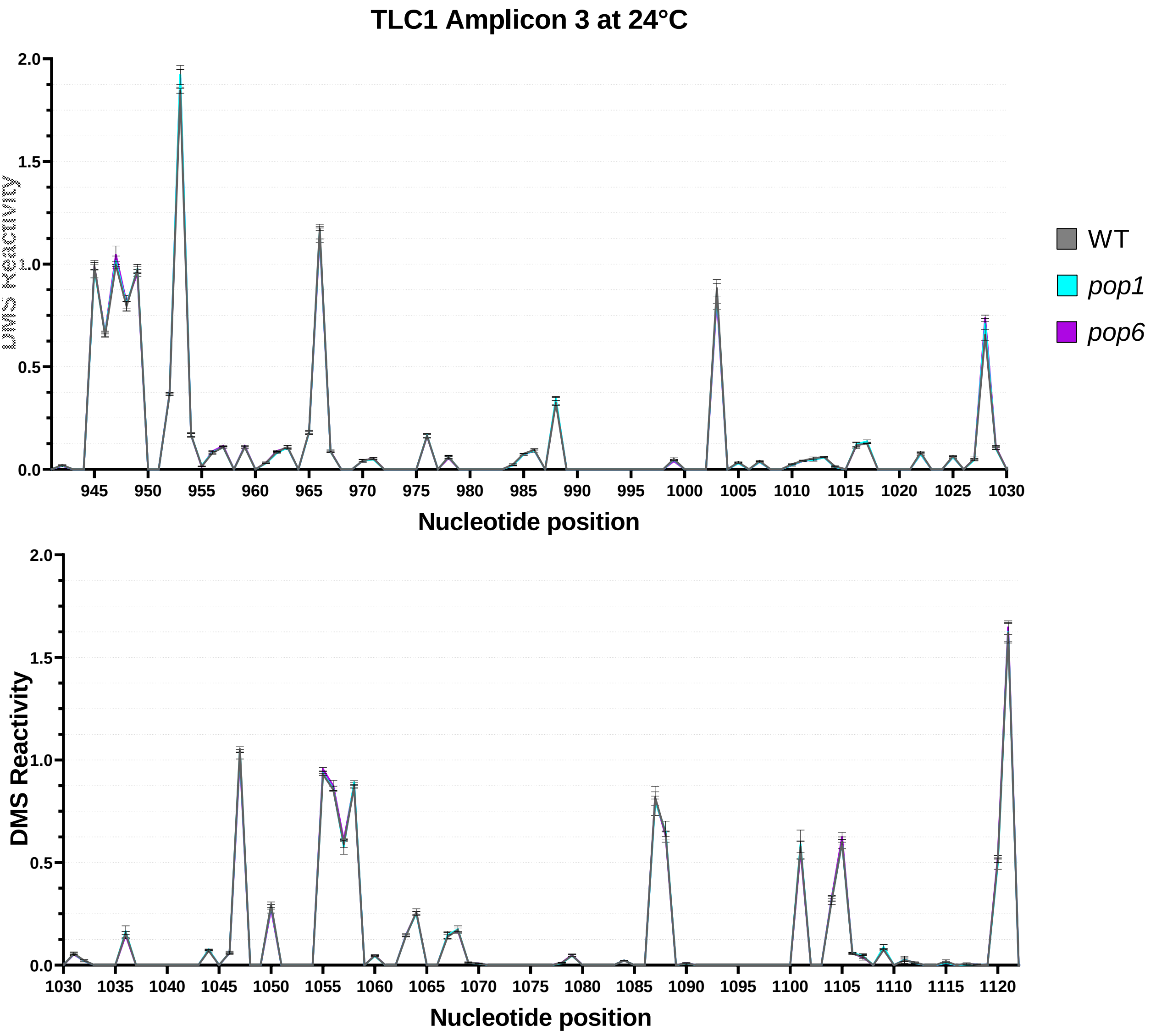

Supplementary Figure 10: DMS reactivity profiles in TLC1 amplicon 3 at 24°C

Normalized DMS reactivity profiles of TLC1 amplicon 3 (nts 941-1120) in cells grown at 24°C for ~50 generations. No regions were found to be statistically significant in this amplicon.

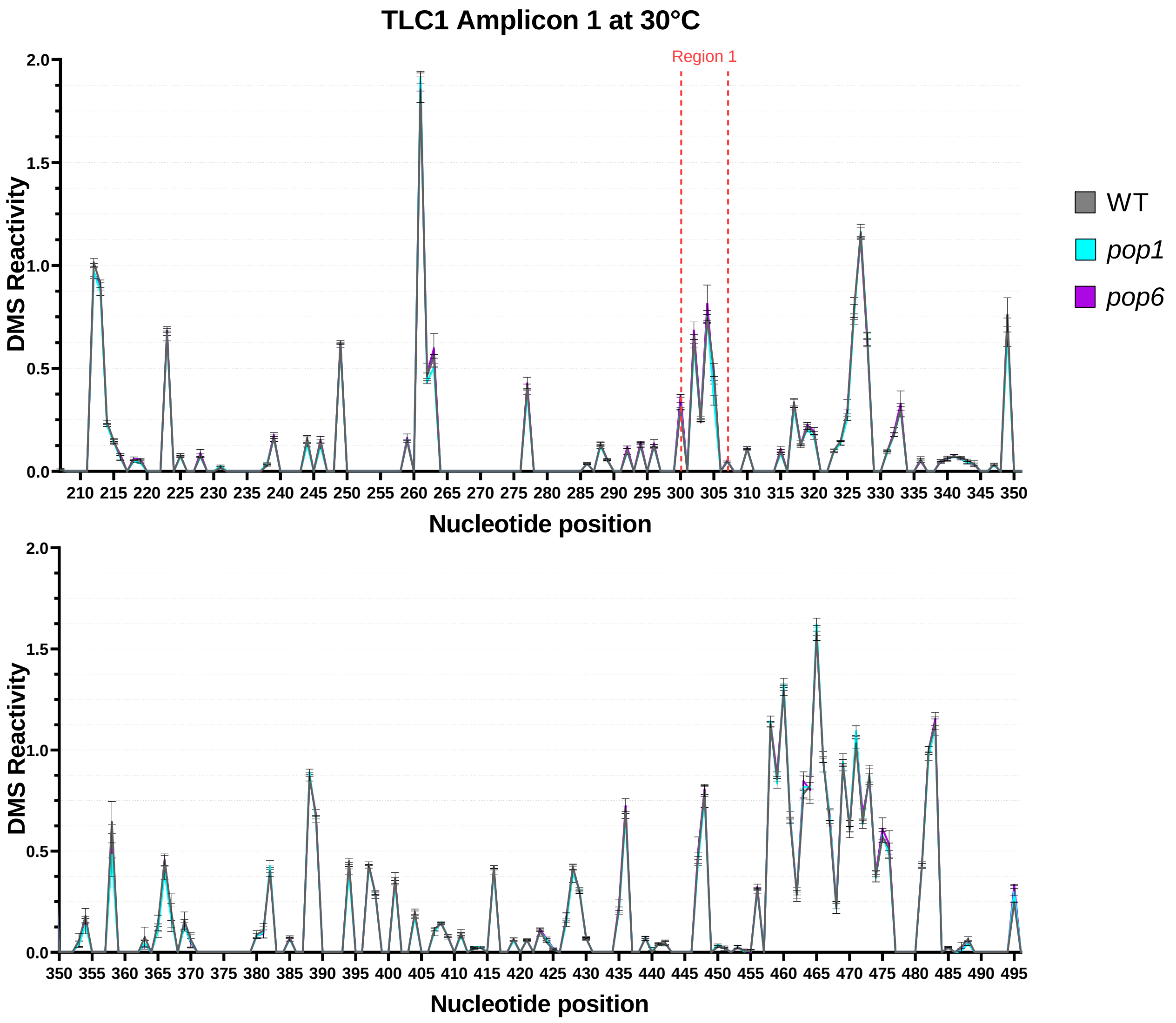

Supplementary Figure 11: DMS reactivity profiles in TLC1 amplicon 1 at 30°C

Normalized DMS reactivity profiles of TLC1 amplicon 1 (nts 212-495) in cells grown at 30°C for ~50 generations. Only one region within this amplicon (indicated by red dashes) had statistically significant differences compared to WT, and this difference was seen only in *pop6* cells. A higher resolution view of the region is shown in Supplementary Fig. 14.

TLC1 Amplicon 2 at 30°C

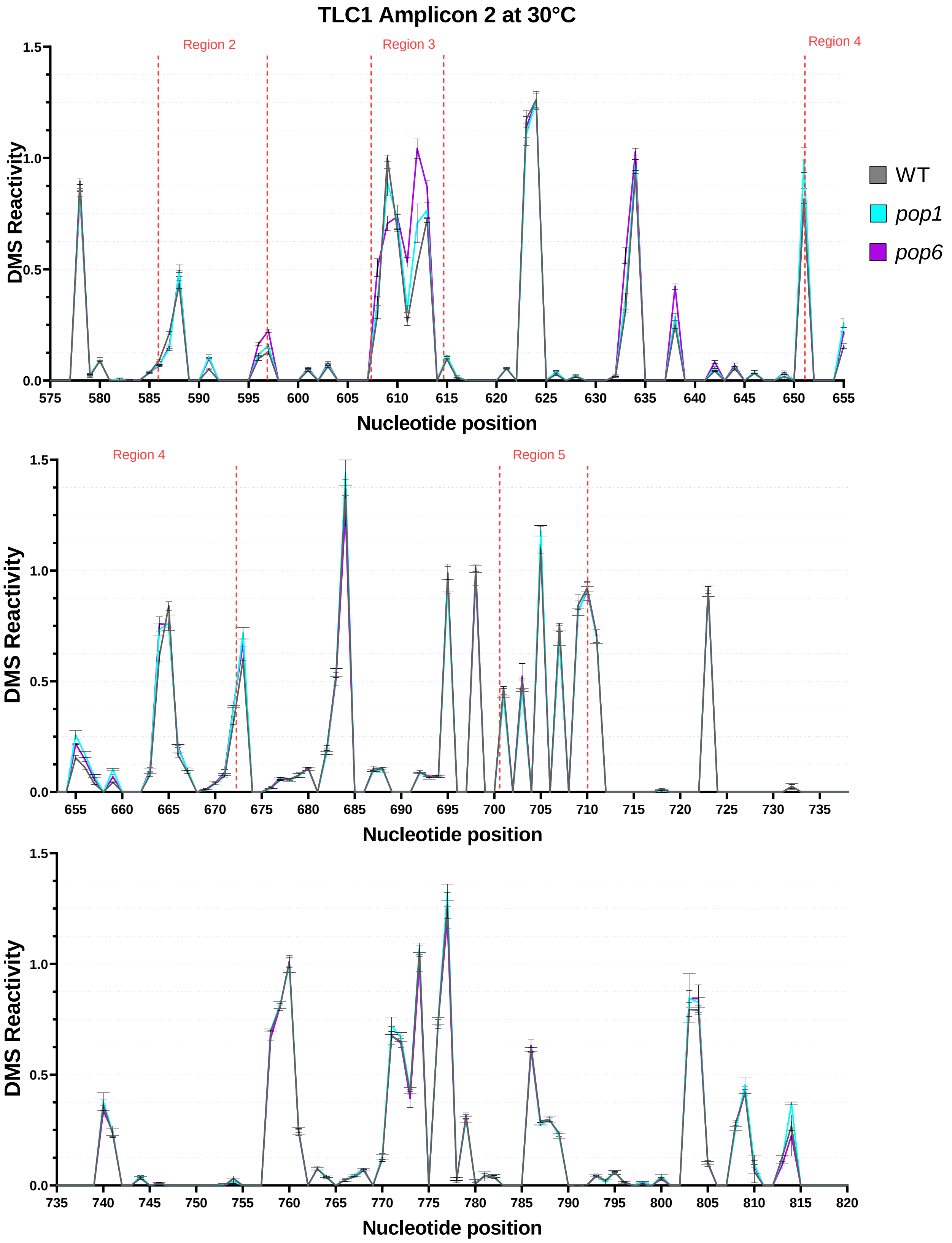

Supplementary Figure 12: DMS reactivity profiles in TLC1 amplicon 2 at 30°C

Normalized DMS reactivity profiles of TLC1 amplicon 2 (nts 578-814) in cells grown at 30°C for ~50 generations. Four regions shown by red dashes had statistically significant differences in *pop* cells compared to WT. A higher resolution view of these regions is shown in Supplementary Fig. 14.

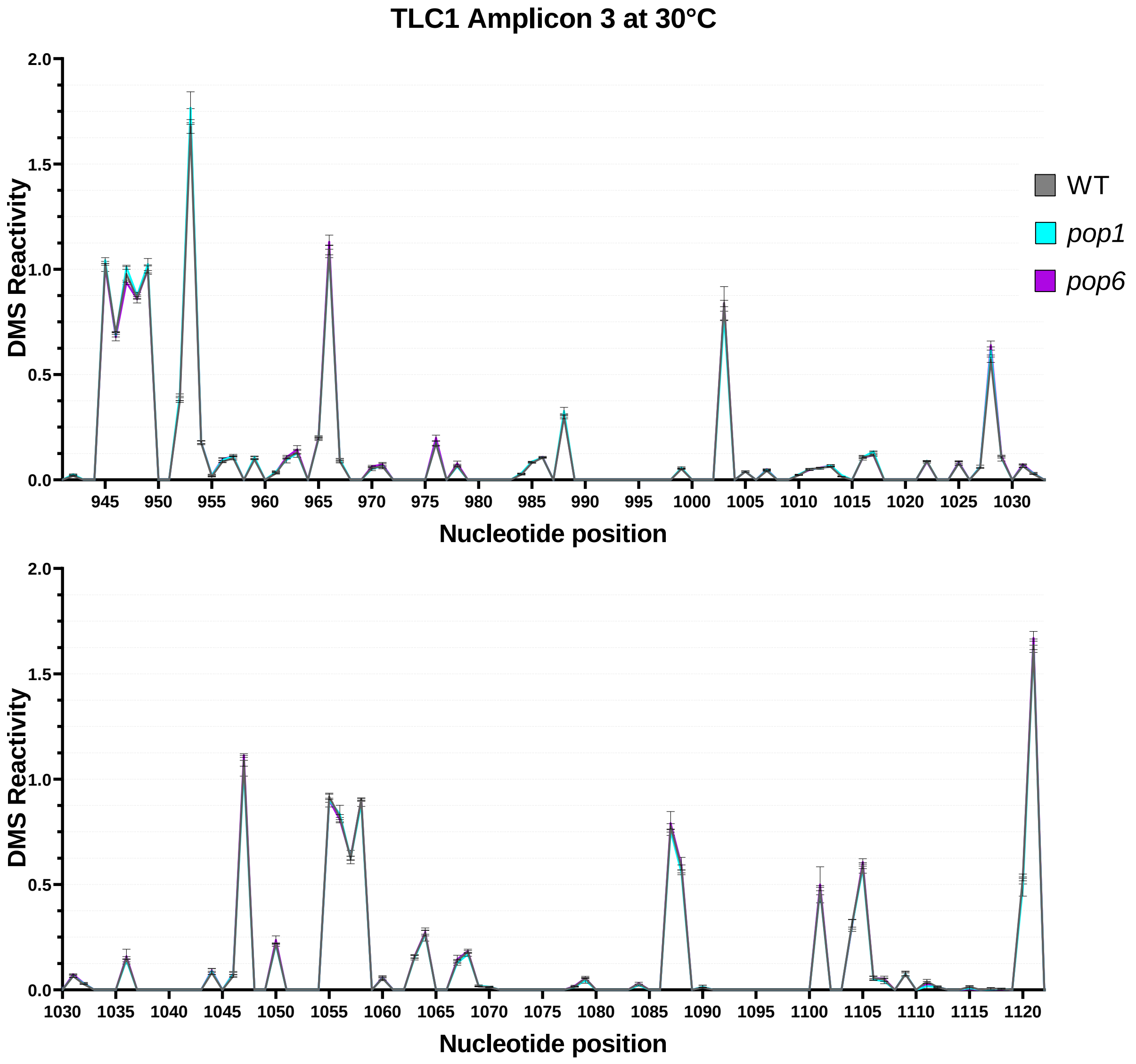

Supplementary Figure 13: DMS reactivity profiles in TLC1 amplicon 3 at 30°C

Normalized DMS reactivity profiles of the TLC1 amplicon 3 (nts 941-1120) in cells grown at 30°C for ~50 generations. There were no regions with statistically significant reactivity differences between *pop* and WT cells in this amplicon at 24 or 30° C.

a

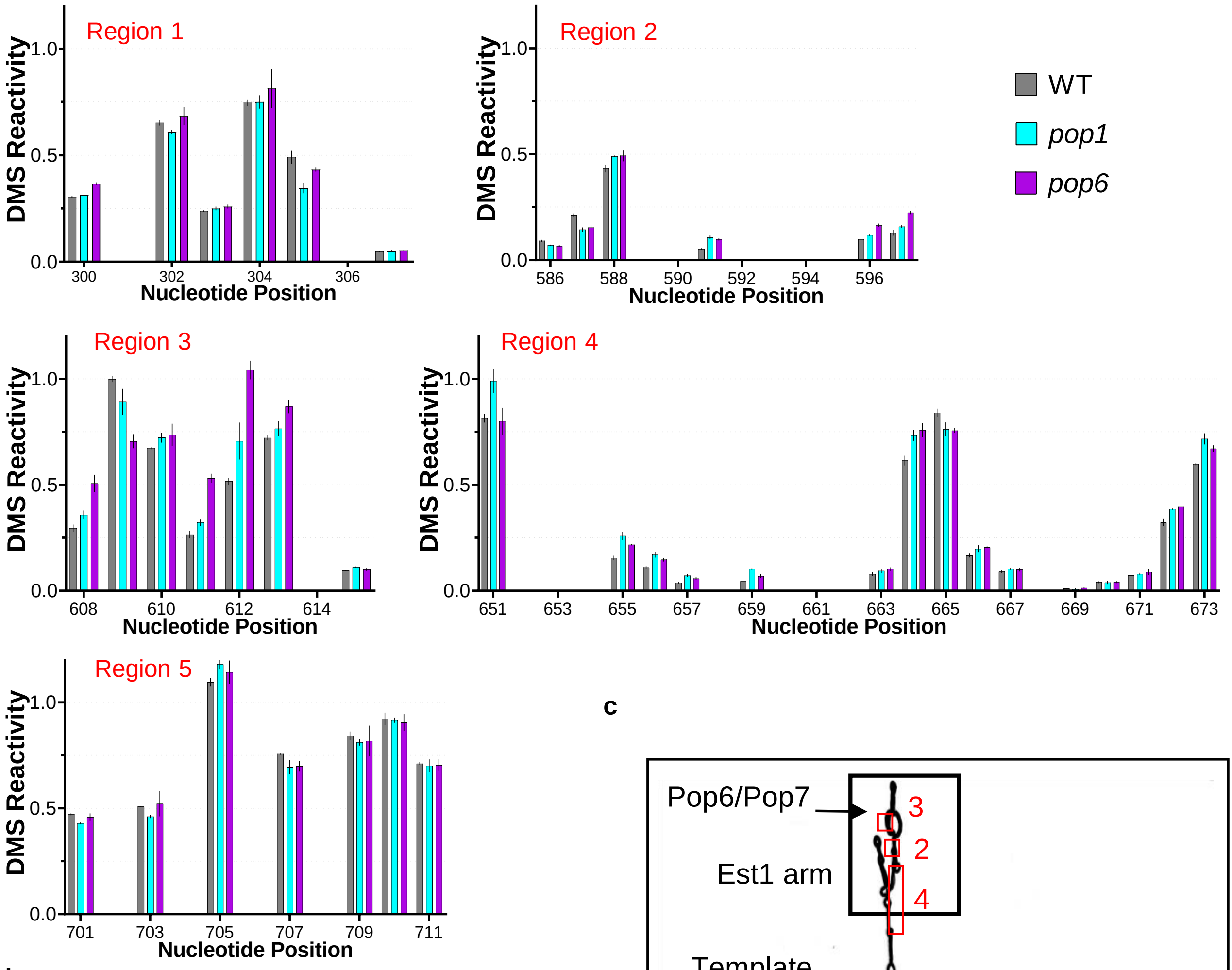

b

q values and Δ*d* values for each indicated region in TLC1

		pop1	***pop6***
**Region 1**	q-value	NS	0.024
	Δ*d*	NS	0.018
**Region 2**	q-value	0.053	0.053
	Δ*d*	0.08	0.11
**Region 3**	q-value	0.024	0.024
	Δ*d*	0.03	0.11
**Region 4**	q-value	0.001	0.02
	Δ*d*	0.06	0.03
**Region 5**	q-value	0.065	NS
	Δ*d*	0.01	NS

c

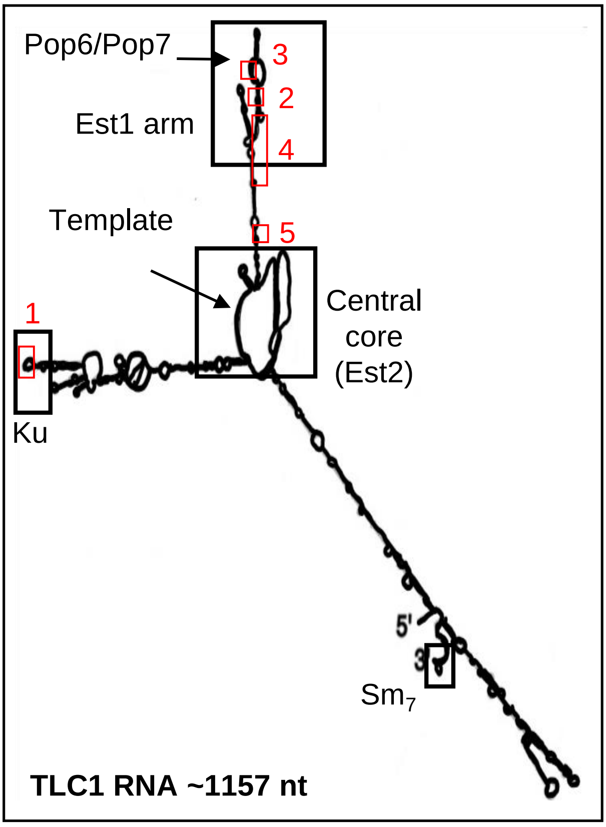

Supplementary Figure 14: TLC1 RNA structure near the Est1 arm is affected in *pop* cells at 30°C.

DMS-MaPseq reactivity analysis from strains grown at 30°C for ~50 generations. **a)** Average normalized DMS reactivity in five regions with statistically significant differential DMS reactivity between the WT and *pop* cells. Error bars indicate the standard deviation from three biological replicates. **b)** Q-values and Δ*d* values of regions 1-5 in *pop1* and *pop6* compared to WT. **c)** The five regions shown in a) are mapped onto the TLC1 RNA structure

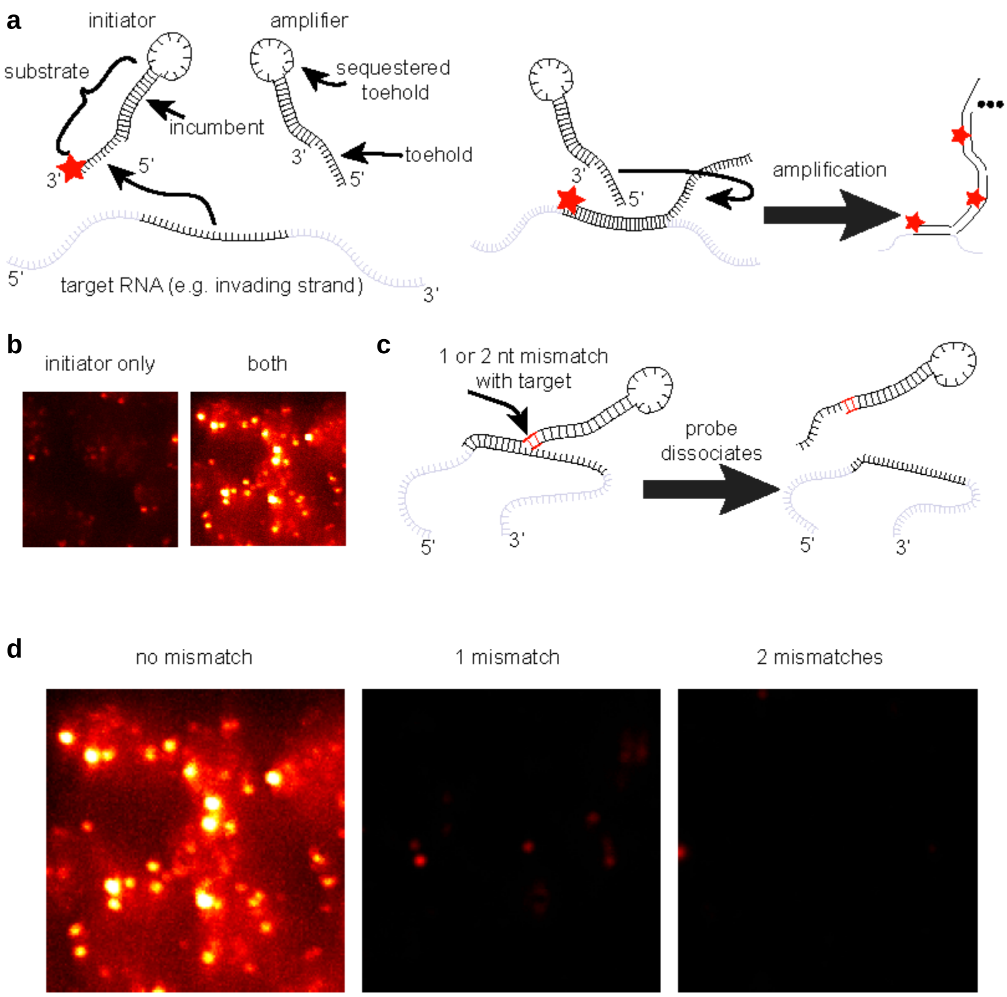

Supplementary Figure 15: Strand displacement-based specificity.

This FISH scheme was previously used to detect yEVenus transcripts with high specificity and signal-to-noise (Wadsworth, et al, 2017). **a)** The proposed mechanism of hairpin probes in hybridization chain reaction (HCR). The hairpin probe consists of a short 10-nt toehold and 16-nt stem with an additional toehold sequestered in a loop. Only a fully matched input RNA can trigger an efficient HCR between amplifier and initiator probes. **b)** Comparison of FISH signal with and without amplification. When the amplifier probe is not present, HCR cannot proceed, and therefore the FISH spots arise from single fluorophores. When the amplifier is present, HCR is initiated, and

the FISH spots become substantially brighter due to colocalization of multiple fluorophores. **c)** Hairpin probe for mismatch discrimination. A fully matching target RNA can displace one of the strands in the hairpin stem, causing stable hybridization of the probe, whereas one or two mismatches between the stem and the target sequences (red) prevent hairpin opening. **d)** The effect of mismatch on FISH signal. With one or two mismatches, the FISH spots disappear almost completely. This comparison demonstrates that hairpin probes can discriminate between single-nucleotide variants.

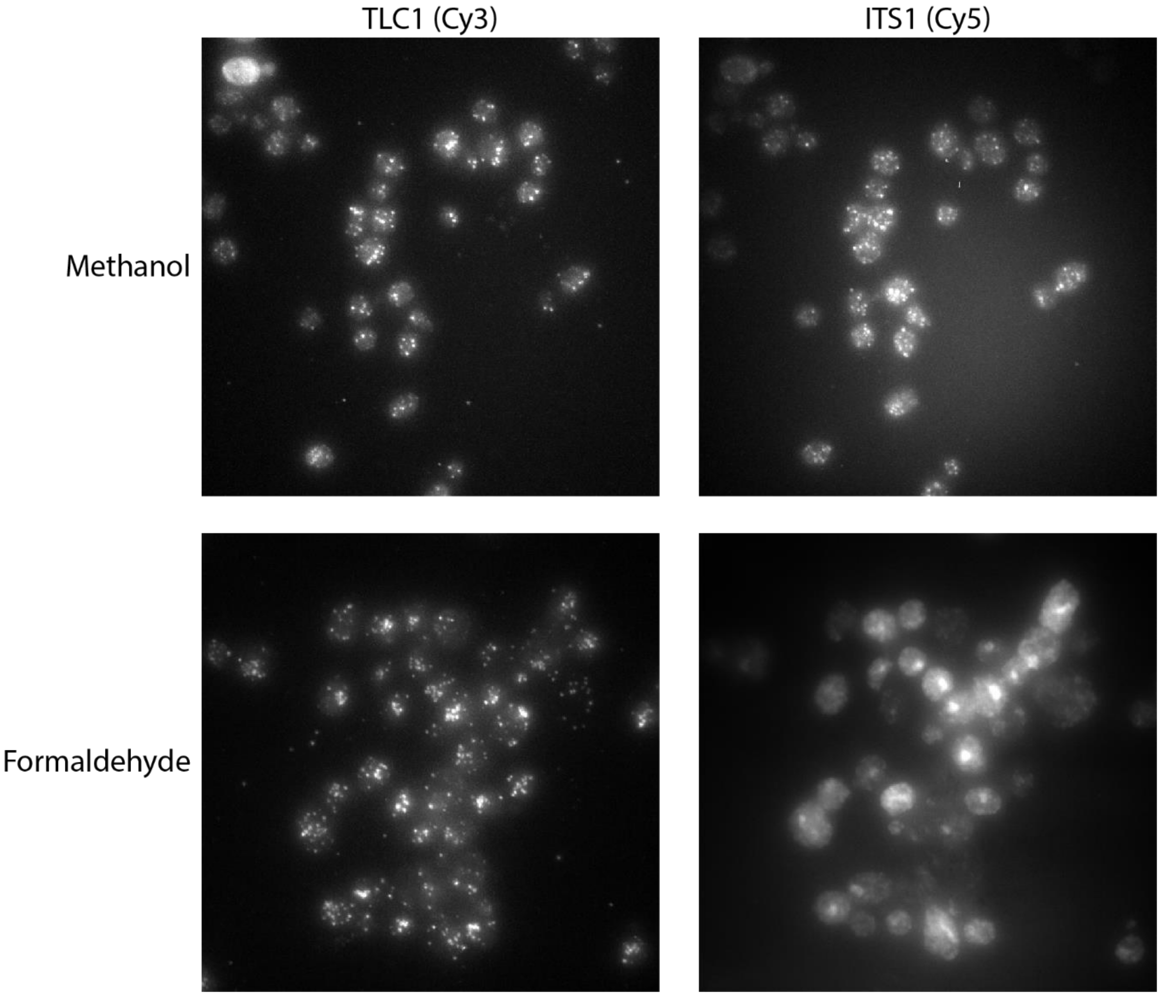

Supplementary Figure 16: Comparison of hairpin probe binding in methanol versus formaldehyde fixed cells.

Wildtype cells were fixed with either methanol (top) or formaldehyde (bottom) and subjected to FISH with the TLC1 (Cy3) and ITS1 (Cy5) probes. The methanol fixed cells show granular and disperse spots in the Cy5 image, whereas the formaldehyde fixed cells show localized high intensity regions. This difference could indicate that the nucleolar structure is disrupted in methanol, but preserved in formaldehyde. The number of TLC1 spots per cell is similar between the two fixatives.

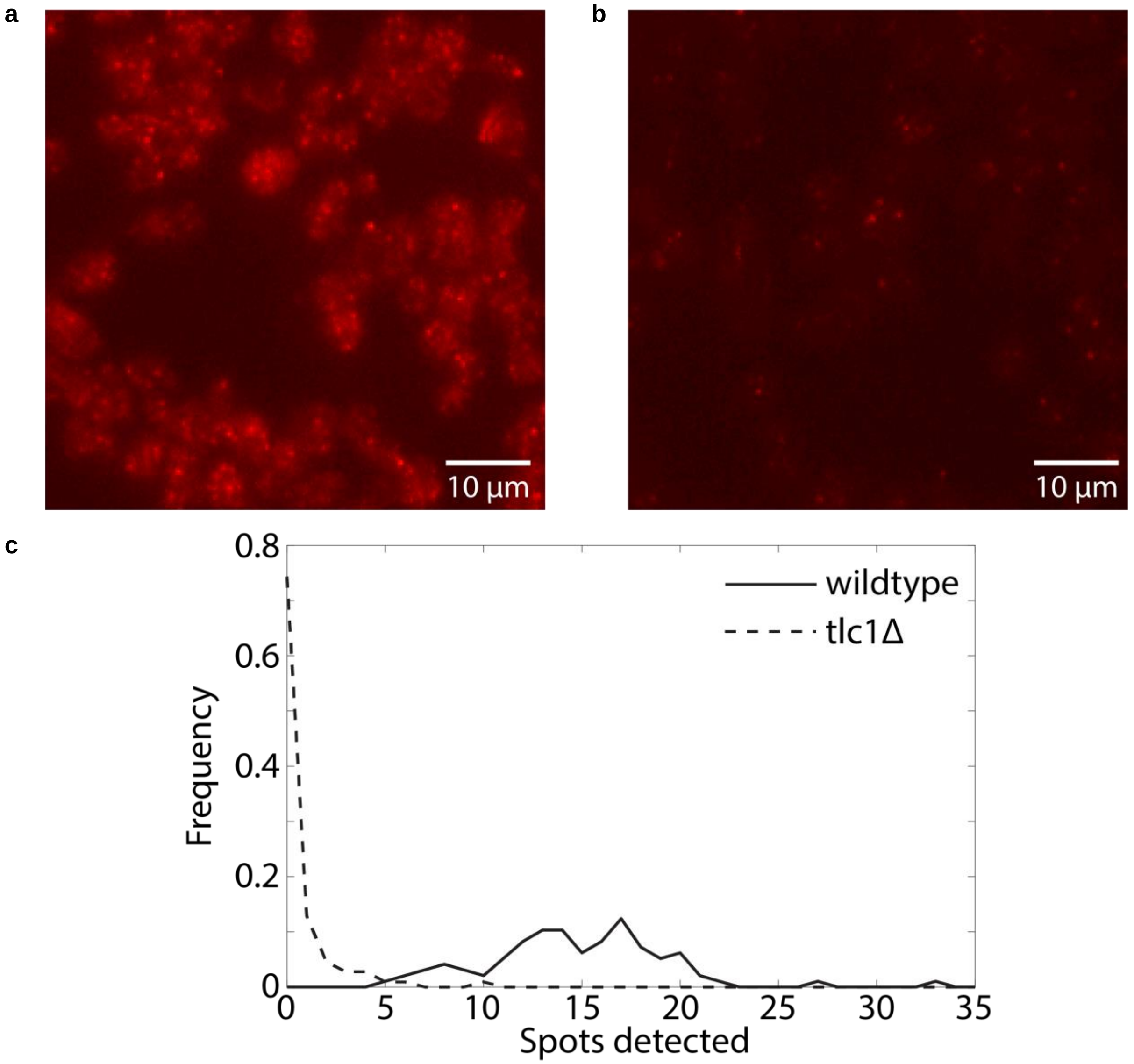

Supplementary Figure 17: TLC1 (Cy3) probe is specific for TLC1 RNA

(a) Sample image of FISH in WT cells using the TLC1 (Cy3) probe. In WT cells, this probe detected 14.69 ± 0.46 spots per cell. Images were acquired in HILO mode on a commercial Andor Dragonfly microscope equipped with 561nm illumination, an Andor Ixon 888 Life camera, and a CFI Apochromat TIRF 60X Nikon objective. **(b)** Sample image of FISH in *tlc1Δ* cells using the TLC1 (Cy3) probe, which detected 0.61±0.14 spots per cell. **(c)** Histograms of the normalized frequency of TLC1 spots in WT and *tlc1Δ* cells.

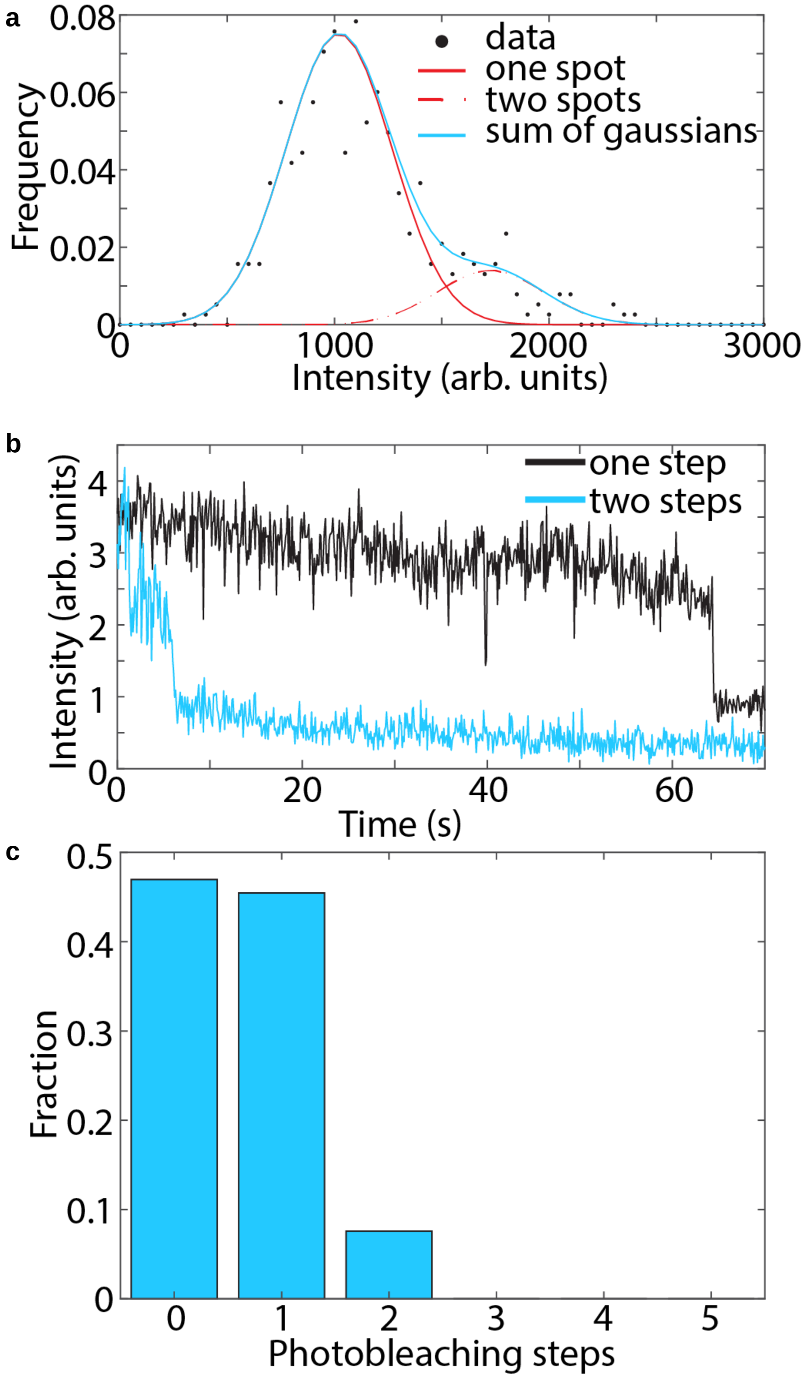

Supplementary Figure 18: Photobleaching of FISH spots using the TLC1 (Cy3) probe.

a) Spot intensity distribution. Spots were measured and intensities were extracted. We used a Gaussian mixture model to infer two populations of spots that arise from a single fluorophore and two fluorophores, respectively.

b) Examples of photobleaching traces. Photobleaching was performed using 100-ms exposure on the Andor Dragonfly microscope. The majority of spots showed one discrete step. **c)** Distribution of the number of photobleaching steps. The majority of photobleaching events occurred in one step (84%). About half of the FISH spots did not show photobleaching steps within five minutes because of the oxygen scavenging system.

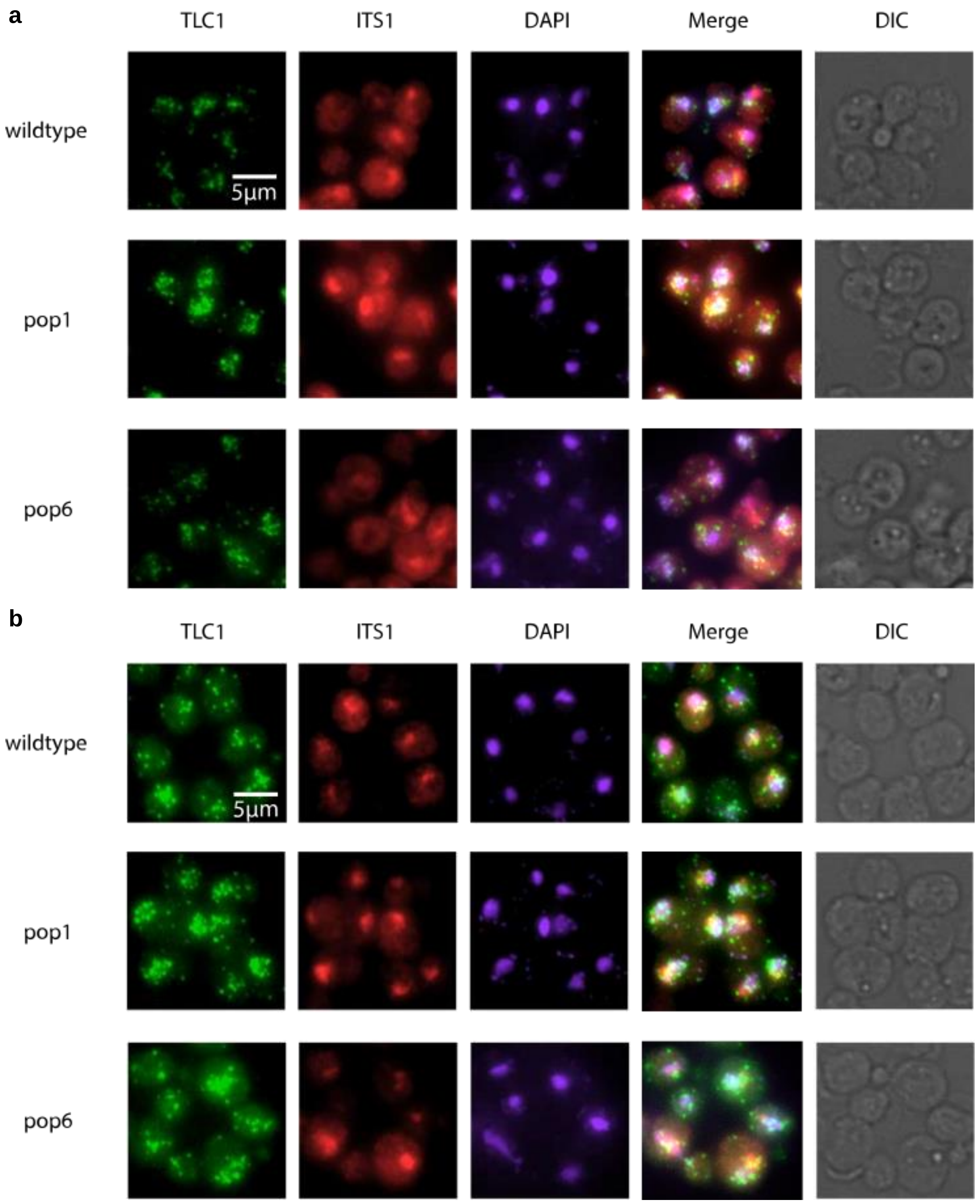

Supplementary Figure 19: Representative cellular images of RNA FISH using the TLC1 (Cy3) probe

2D projection images of cells. **a)** Cells of indicated genotypes were grown at 24°C (permissive growth). Images in each channel are scaled to the same contrast. **b)** Cells of indicated genotypes were grown at 30°C (semi-permissive growth). Images in a) and b) are scaled to the same contrast.

Supplemental Table 1. Yeast strains

Strain name	Genotype	Source
yDG199	S288c Mat a *his3Δ1 leu2Δ0 ura3Δ0 met15Δ0*	Costanzo, M. *et al.* 2016
yDG187	S288c Mat a *his3Δ1 leu2Δ0 ura3Δ0 met15Δ0 lys2Δ0 pop6-502::KanR*	Costanzo, M. *et al.* 2016
yDG190	S288c Mat a *his3Δ1 leu2Δ0 ura3Δ0 met15Δ0 lys2Δ0 pop1-500::KanR*	Costanzo, M. *et al.* 2016
yDG270	S288c Mat a *his3Δ1 leu2Δ0 ura3Δ0 met15Δ0 lys2Δ0 pop1-500::KanR pRS416-POP1:URA3*	This study
yDG272	S288c Mat a *his3Δ1 leu2Δ0 ura3Δ0 met15Δ0 lys2Δ0 pop1-500::KanR pRS416-URA3*	This study
yDG282	S288c Mat a *his3Δ1 leu2Δ0 ura3Δ0 met15Δ0 lys2Δ0 pop6-502::KanR pRS416-POP6:URA3*	This study
yDG284	S288c Mat a *his3Δ1 leu2Δ0 ura3Δ0 met15Δ0 lys2Δ0 pop6-502::KanR pRS416-URA3*	This study
yDG443	S288c Mat a, *his3Δ1 leu2Δ0 ura3Δ0 met15Δ0 EST2-*Gly_8*-13Myc::HIS3MX6*	This study
yDG446	S288c Mat a *his3Δ1 leu2Δ0 ura3Δ0 met15Δ0 lys2Δ0 pop1-500::KanR EST2-*Gly_8-13Myc::HIS3MX6	This study
yDG450	S288c Mat a *his3Δ1 leu2Δ0 ura3Δ0 met15Δ0 lys2Δ0 pop6-502::KanR EST2-*Gly_8-13Myc::HIS3MX6	This study
yDG454	S288c Mat a, his3Δ1 leu2Δ0 ura3Δ0 met15Δ0 *EST1-*Gly_8-13Myc::HIS3MX6	This study
yDG457	S288c Mat a *his3Δ1 leu2Δ0 ura3Δ0 met15Δ0 lys2Δ0 pop1-500::KanR EST1-*Gly_8-13Myc::HIS3MX6	This study
yDG460	S288c Mat a *his3Δ1 leu2Δ0 ura3Δ0 met15Δ0 lys2Δ0 pop6-502::KanR EST1-*Gly_8-13Myc::HIS3MX6	This study
yDG482	S288c Mat a/α *his3Δ1 leu2Δ0 ura3Δ0 met15Δ0 ku70Δ::HIS3*	This study
yDG483	S288c Mat a/α *his3Δ1 leu2Δ0 ura3Δ0 met15Δ0 ku70Δ::HIS3 pop1-500::KANMX*	This study
yDG484	S288c Mat a/α *his3Δ1 leu2Δ0 ura3Δ0 met15Δ0 ku70Δ::HIS3 pop6-502::KANMX*	This study
yDG538	S288c Mat a *his3Δ1 leu2Δ0 ura3Δ0 met15Δ0 3HA-POP1::HIS3MX6*	This study
yDG542	S288c Mat a *his3Δ1 leu2Δ0 ura3Δ0 met15Δ0 3HA-pop1-500::HIS3MX6*	This study
yDG547	S288c Mat a *his3Δ1 leu2Δ0 ura3Δ0 met15Δ0 3HA-POP1::HIS3MX6 pop6-502*	This study

Supplemental Table 2. Plasmids

Name	Genotype	Source
pDG1	pRS416 URA3, CEN	Sikorski 1989
pDG2	pRS416 URA3, CEN, POP1	This study
pDG3	pRS416 URA3, CEN, POP6	This study

Supplemental Table 3. Primers

DG52	GTAACATCGTTATGTCCGG TGGTAC	Act1. Fwd for RT-qPCR
DG53	CCAAGATAGAACCACCAAT CCAGAC	Act1, rev for RT-qPCR
DG192	GGA ATT CCC TCG AGA AGT TAA GAA ATA AAG AGA AAG TCA AAA AAG AAA AGA CTA GTA AG	Fwd, for amplifying and cloning POP1 into URA3 CEN plasmids (pRS416) XhoI and HindIII
DG193	AAC TGC AGA ACC AAA GCT TGA ACT TCT TCT CTC TAT TTA TAC GTC TTG CCA ATG GAA AAC	Rev, for amplifying and cloning POP1 into URA3 CEN plasmids (pRS416) XhoI and HindIII
DG194	GGA ATT CCC TCG AGC TTT TCT TTT TTG TTG GCA GTT TCA AGC AAC ACA TGT AC	Fwd, for amplifying and cloning POP6 into URA3 CEN plasmids (pRS416) XhoI andHindIII
DG195	AAC TGC AGA ACC AAA GCT TGG AAC AAT TGG TAG TTC TGA TAT CGA TTA AGC CTT C	Rev, for amplifying and cloning POP6 into URA3 CEN plasmids (pRS416) XhoI and HindIII
DG330	CAG TAA TAT GCT TTG GGT TG	NME1, fwd RT-qPCR primer
DG331	GTA AGC TCC ATT GGG TTA CT	NME1, rev RT-qPCR primer
DG293	AAT GGC TGT TGC GTT TGC TT	Fwd, new primers to check TLC1 by RT-qPCR
DG294	ACC ACA AAT TGC GCA CAC AC	Rev, new primers to check TLC1 by RT-qPCR
DG298	AACTATCACTGGCCCACTA TCCTCTGATTTTCTCTCATA TCCAGACGAAGCAATTGAT GCTGATGAGGACATCACCG TCCAAGTGCCAGATACTCC TACTGGTGGTGGTGGAGG TGGAGGTGGTCGGATCCC CGGGTTAATTAA	Fwd, to amplify G8-13Myc::HIS3 from pFA6a from Longtine plamid series. Homology to Est1

Supplemental Table 3. Primers

DG299	CCCCATATTTTCGTGGGTTGACGACGACAGGCGTGGACGACGTTAATTGACGTCGTCATAATATATTTCATATTATGATTTTTTCCCTCACCATTACTTGTTCTCgaattcgagctcgtttaaac	Rev, to amplify G8-13Myc::HIS3 from pFA6a from Longtine plamid series. Homology to Est1
DG300	ATCTTCAAACACATCAAAATTTAAAGATAATATCATTCTTTTGAGAAAGGAAATTCAACACTTGCAAGCATATATATATATATATATACATATAGTTAATGGTGGTGGTGGAGGTGGAGGTGGTCGGATCCCCGGGTTAATTAA	Fwd, to amplify G8-13Myc::HIS3 from pFA6a from Longtine plamid series. Homology to Est2
DG301	GCTAACGGAAAAGAGATTAGTGCTTTTGGCTTATTACAAAGTTTGCGGCAATATTTTCCTTATCAGCATCATAAGCTGTCAGTATTTCATGTATTATTAGTAGAATTCGAGCTCGTTTAAAC	Rev, to amplify G8-13Myc::HIS3 from pFA6a from Longtine plamid series. Homology to Est2
DG348	TCGTCGGCAGCGTC tgatctttcagttgatagcctgctgctcttt	Fwd, TLC1 amplicon 1. DMS-MaPseq
DG365	GTCTCGTGGGCTCGG ACAGCGAACTCGTGCAAACTTTGCT	Rev, TLC1 amplicon 1. DMS-MaPseq
DG364	TCGTCGGCAGCGTC CTCGTTTTCTTATACCTAGTATTTTTTCTGACACTGTTTAAGGTG	Fwd, TLC1 amplicon 2. DMS-MaPseq
DG351	GTCTCGTGGGCTCGG TTCGATGTACGGGGCACATTTGGAAATTTCATCA	Rev, TLC1 amplicon 2. DMS-MaPseq
DG352	TCGTCGGCAGCGTCcggttaggtttgcgggcgatcagtaa	Fwd, TLC1 amplicon 3. DMS-MaPseq

Supplemental Table 3. Primers

DG353	GTCTCGTGGGCTCGG AAATGTTTCCAAAAATTAT CTAAATGCATCGAAGGCA	Rev, TLC1 amplicon 3. DMS-MaPseq
DG354	TCGTCGGCAGCGTC AATCCATGACCAAAGAATC GTCACAAATCG	Fwd, NME1. DMS-MaPseq
DG355	GTCTCGTGGGCTCGG CCATTGGGTTACTCGATCC AGCACG	Rev, NME1. DMS-MaPseq
DG358	TAA CCC TGT CCA ACC TGT CT	XV-L fwd. Telomere sequence for qPCR. ChIP
DG359	ATA CTA TAG CAT CCG TGG GC	XV-R rev. Telomere sequence for qPCR. ChIP
DG360	ATC ATT GAG GAT CTA TAA	VI-R fwd. Telomere sequence for qPCR. ChIP
DG361	CTT CAC TCC ATT GCG	VI-R rev. Telomere sequence for qPCR. ChIP
DG362	TCG TTA CAA GGT GAT G	ARO1. ChiP control
DG363	AAT AGC GGC AAC AAC	ARO1. ChIP control
GW1	/5Cy3/GGATCGGTACGAAG AAGGAATAACAGTAGAAA GGTACTGTTATTCCTTCTTC	TLC1 probe. FISH RNA
GW2	GGATCGGTACGAAGAAGG AATAACAG	TLC1control unlabeled probe. FISH RNA
GW3	/5Cy5/GCCCGGCTGGACTC TCCATCTCTTGTCTTCAGA TTGAACGGAAGACAAGAG ATGGAGAGT	ITS RNA. Probe
GW4	GCCCGGCTGGACTCTCCAT CTCTTGTCTTC	ITS control unlabeled probe
UP1	CTACGTAACGATTGATGGT GCCTACAG	Universal reverse transcription primer – 3’RACE (Deng et al, NAR, 2018).
DG389	TCAAGAACGTAATTTGAGA TTTTTCAAGATGG	TLC1. Internal Primer 3’RACE
