# Supplementary material for: Stability and nuclear localization of yeast telomerase depend on protein components of RNase P/MRP
Source: Nat Commun. 2020 May 1;11:2173. doi: 10.1038/s41467-020-15875-9 (PMC7195438; doi:10.1038/s41467-020-15875-9)
Supplement: Supplementary file 3 — Reporting Summary [file 41467_2020_15875_MOESM3_ESM.pdf]

nature research

Corresponding author(s): Virginia A. Zakian

Last updated by author(s): 3/3/2020

Reporting Summary

Nature Research wishes to improve the reproducibility of the work that we publish. This form provides structure for consistency and transparency in reporting. For further information on Nature Research policies, see Authors & Referees and the Editorial Policy Checklist.

Statistics

For all statistical analyses, confirm that the following items are present in the figure legend, table legend, main text, or Methods section.

n/a	Confirmed	
☐	☒	The exact sample size (*n*) for each experimental group/condition, given as a discrete number and unit of measurement
☐	☒	A statement on whether measurements were taken from distinct samples or whether the same sample was measured repeatedly
☐	☒	The statistical test(s) used AND whether they are one- or two-sided<br>*Only common tests should be described solely by name; describe more complex techniques in the Methods section.*
☐	☒	A description of all covariates tested
☐	☒	A description of any assumptions or corrections, such as tests of normality and adjustment for multiple comparisons
☐	☒	A full description of the statistical parameters including central tendency (e.g. means) or other basic estimates (e.g. regression coefficient) AND variation (e.g. standard deviation) or associated estimates of uncertainty (e.g. confidence intervals)
☐	☒	For null hypothesis testing, the test statistic (e.g. *F*, *t*, *r*) with confidence intervals, effect sizes, degrees of freedom and *P* value noted<br>*Give P values as exact values whenever suitable.*
☒	☐	For Bayesian analysis, information on the choice of priors and Markov chain Monte Carlo settings
☒	☐	For hierarchical and complex designs, identification of the appropriate level for tests and full reporting of outcomes
☒	☐	Estimates of effect sizes (e.g. Cohen's *d*, Pearson's *r*), indicating how they were calculated

Our web collection on statistics for biologists contains articles on many of the points above.

Software and code

Policy information about availability of computer code

Data collection	Data collection is described in Methods and code is available at https://github.com/hepcat72/RNAseP-MRP/ and described in Methods
Data analysis	Software is available at https://github.com/hepcat72/RNAseP-MRP/ and described in Methods

For manuscripts utilizing custom algorithms or software that are central to the research but not yet described in published literature, software must be made available to editors/reviewers. We strongly encourage code deposition in a community repository (e.g. GitHub). See the Nature Research guidelines for submitting code & software for further information.

Data

Policy information about availability of data

All manuscripts must include a data availability statement. This statement should provide the following information, where applicable:

- Accession codes, unique identifiers, or web links for publicly available datasets
- A list of figures that have associated raw data
- A description of any restrictions on data availability

The 3' RACE and DMS-MaPseq datasets generated and analyzed during the current study are available in the PRJNA548560 and PRJNA548768 repositories, respectively. Persistent web links https://www.ncbi.nlm.nih.gov/sra/PRJNA548560 and http://www.ncbi.nlm.nih.gov/bioproject/548768

Field-specific reporting

Please select the one below that is the best fit for your research. If you are not sure, read the appropriate sections before making your selection.

☒ Life sciences ☐ Behavioural & social sciences ☐ Ecological, evolutionary & environmental sciences

Life sciences study design

All studies must disclose on these points even when the disclosure is negative.

Sample size	At least 3 biological replicates of each strain were used in all experiments, except for the 3' RACE assay (2 biological replicates). Based on previous studies, the read coverage and high reproducibility between biological replicates was deemed sufficient for this 3' RACE assay.
Data exclusions	No data was excluded in this study.
Replication	All replication attempts were successful in this study.
Randomization	All WT and mutant strains were grown and treated equally in all the experiments described in this study.
Blinding	Blinding is not relevant in this study. Our goal is to understand the differences between WT and pop mutant strains.

Reporting for specific materials, systems and methods

We require information from authors about some types of materials, experimental systems and methods used in many studies. Here, indicate whether each material, system or method listed is relevant to your study. If you are not sure if a list item applies to your research, read the appropriate section before selecting a response.

Materials & experimental systems

n/a	Involved in the study	
☐	☒	Antibodies
☒	☐	Eukaryotic cell lines
☒	☐	Palaeontology
☒	☐	Animals and other organisms
☒	☐	Human research participants
☒	☐	Clinical data

Methods

n/a	Involved in the study	
☒	☐	ChIP-seq
☒	☐	Flow cytometry
☒	☐	MRI-based neuroimaging

Antibodies

Antibodies used	c-MYC antibody (Takara 631206), monoclonal HA antibody (Santa cruz-sc-7392), PGK1 (Abcam), anti-α-Tubulin antibody (Sigma T6074), anti-2,2,7-Trimethylguanosine Mouse mAb (K121) Agarose Conjugate (Millipore)
Validation	Validation of antibodies was done by Western blot analysis in this study and/or by the manufacturer.
